# Burnout and associated risk factors among nurses working in COVID-19 isolation hospitals: a cross-sectional study in Egypt

**DOI:** 10.1186/s42506-025-00192-0

**Published:** 2025-05-12

**Authors:** Ola G. Ibraheim, Abdallah I. Shehata, Taghareed A. Elhoseny

**Affiliations:** 1 Directorate of Health Affairs, Alexandria, Egypt; 2 Department of Health Administration and Behavioural Sciences, High Institute of Public Health, University of Alexandria, Alexandria, Egypt

**Keywords:** Burnout, COVID-19, Maslach Burnout Inventory, Occupational stress, Mental health, Healthcare workforce, Staff well-being, Nurses

## Abstract

**Background:**

Nurses are vulnerable to burnout due to the high risk and pressure associated with their job performance. The coronavirus disease of 2019 (COVID-19) pandemic imposed an additional demand and stress on the healthcare workforce worldwide. This study aimed to assess the magnitude of burnout syndrome among nurses working in COVID-19 isolation hospitals and its associated factors.

**Methods:**

A cross-sectional study was conducted among 385 nurses working in two COVID-19 isolation hospitals between April and August 2022 in Alexandria, Egypt. A predesigned self-administered questionnaire including questions about sociodemographic and work-related factors and an Arabic-translated version of Maslach Burnout Inventory Human Services Survey "MBI-HSS" was used.

**Results:**

Of the total sample, 82.3% had high levels of emotional exhaustion (EE) with a mean score of 35.43 ± 9.52 (The range of the subscale score for EE = 0–54), 60.8% had a high level of depersonalization (DP) with a mean of 13.63 ± 5.81 (The range for DP = 0–30), 34.5% had a low level of personal accomplishment (PA) with a mean score of 33.70 ± 7.43 (The range for PA = 0–48 (reversed). Nurses having no children, nurses with higher educational level, and those who reported inadequacy of the number of physicians had significantly higher odds (3.98, 2.37, and 3.25, respectively) of having a high level of emotional exhaustion. Nurses having no children, and those who reported inadequacy of the number of physicians in their units had significantly higher odds (2.4 and 2.2) of having a high level of depersonalization.

**Conclusion:**

Nurses working in COVID-19 isolation hospitals had high levels of burnout syndrome. Predictors of EE or DP included having no children, higher levels of education, and reporting an inadequate number of physicians. Stress coping training and psychological and social support services are recommended to overcome the relevant stresses and burnout provoking factors.

## Introduction

The nursing profession is among the most common jobs suffering from burnout. Maslach described burnout as a condition that results from chronic interpersonal stressors within one’s job [1]. Burnout has been classified within the International Classification of Diseases as an occupational phenomenon rather than a medical condition [2]. A well-known definition of burnout describes it as a syndrome of emotional exhaustion, depersonalization, and reduced personal accomplishment [3]. Most studies that measure burnout examine those three key dimensions using the Maslach Burnout Inventory (MBI), one of the most familiar valid tools [4]. 

Research on burnout syndrome is widespread, and it has shown that job characteristics such as high workload, time pressure, long work hours, and focusing on people have been defined as predisposing factors. All of them are known to be characteristic of the profession of nursing [3, 5]. The prevalence of burnout has been reported to be high among nurses all over the world [6–9]. A meta-analysis of 94 studies about the prevalence of burnout among nurses covering 30 countries between 2012 and 2022 showed a pooled prevalence of 30% [10]. In the Middle East, a systematic review of 53 burnout studies among healthcare workers in seven countries including Egypt was carried out and included 10,992 nurses. It has shown that nurses, along with other healthcare workers, had high levels of burnouts ranging from 21.7 to 37.9%. The mean emotional exhaustion score ranged from 13.1 ± 6.6 to 48.9 ± 6.1, depersonalization score from 2.6 ± 31 to 28.5 ± 9.1 and reduced personal accomplishment from 2.6 ± 0.5 to 47.9 ± 13.7 [11]. 

In Egypt, high levels of burnout among nurses have been shown in university hospitals. A comprehensive study involving 286 nursing staff conducted at Beni-Suef University in 2016, found that about 43.2% were classified as having a moderate level of burnout and 32.6% as having a high level of burnout; 54.6% of nurses had average levels of emotional exhaustion, 48% scored high on depersonalization, and 77.5% had high levels of reduced personal accomplishment [9]. In another university hospital in Tanta in 2017, studying burnout among nurses using MBI showed that 52.8% had a high level of emotional exhaustion, 44.4% had a moderate level of depersonalization, and 96.5% had a high level of reduced personal accomplishment [12]. 

Factors associated with or predicting burnout have been widely studied among nurses to modify and alleviate those factors. Personal characteristics such as gender, age, education level, work years, and family status were shown to be related to burnout [7, 8, 12–14]. Job characteristics such as longer work hours, irregular shifts, short experience, larger hospital size, high workload, low schedule flexibility, managerial position, and negative nurse-physician relationship were all shown to be either associated or significantly associated with one or more of the burnout dimensions [12, 14–16]. 

The COVID-19 pandemic added to the stressful environment of nursing. Being the frontline providers of healthcare and at the same time the majority of staff, nurses suffered burnout to a higher degree during this global crisis [17]. In a study by Rathnayake et al. in Sri Lanka, nurses mentioned that fear of contracting COVID-19 illness and spreading it to their families and the community, and being rejected by peers, coworkers, family members, neighbors, and society left them frustrated. Witnessing patients'feelings such as fear of dying, wearing personal protection equipment (PPE) for prolonged hours, and insufficient rest placed more stress on nurses [18]. Such stresses could be a factor that exacerbates the burnout among nurses.

In Egypt, studies on nurses during COVID-19 reported high levels of burnout. In Benha University Hospital in 2020, burnout was detected among 64% of nurses using an Arabic translation of the MBI [19]. In Port Said during the same year, the level of burnout among 250 nurses working in primary healthcare facilities using the Arabic version of MBI was 86% [20]. Compared with other healthcare settings, in COVID-19 isolation hospitals where cases were referred and treated, healthcare workers, especially nurses, are at a higher risk of exposure to the virus [19, 20]. This added stress to nurses may be a possible factor that worsens the situation. This study aimed to assess burnout and the associated personal and work-related factors among nurses working in two isolation hospitals in Alexandria, Egypt during the COVID-19 pandemic. 

## Methods

### Study setting

This study was conducted at two public hospitals that were used as COVID-19 isolation hospitals during the pandemic in Alexandria, Egypt. Both are specialized hospitals (fever and chest) and are affiliated with the Ministry of Health and Population (MOHP). The first is a 265-bed hospital and the second is a 209-bed hospital. During the pandemic, the Fever and Chest hospitals were used for the initial examination and referral of cases then they were assigned as isolation hospitals too. Among other isolation hospitals these two were selected based on their specialty, large size, high number of nurses, and feasibility of obtaining administrative consent.

### Study design

A cross-sectional study design was used.

### Target population

Nurses working in the COVID-19 isolation hospitals. The study population was nurses working in the first hospital (*n* = 290) and the second hospital (*n* = 265).

### Sample size and sampling technique

Assuming that 50% is the expected proportion of nurses suffering from burnout in Alexandria isolation hospitals during the COVID-19 pandemic, with a confidence level of 95% and a degree of precision of 5%, the minimum required sample size is 384. This number was rounded to 385. This assumption of prevalence was made to yield an adequate sample size, as no data on burnout among nurses during COVID-19 in isolation hospitals were available at that time.

#### Method of selection

 A convenience sampling technique was used as all nurses working at the start of data collection in the selected hospitals who agreed to participate were included till the completion of the required sample size from April to August 2022. All shifts and units were visited equally. In total, 200 nurses from the first hospital and 185 nurses from the second hospital were included.

### Study tools

A predesigned self-administered questionnaire was used for data collection. The first section included questions about sociodemographic data such as age, gender, marital status, level of education, number of children, years of experience, adequacy of income, as well as work-related data such as service area/unit, nurse-to-patient ratio, working shift, working hours per week, adequacy of personal protective equipment and adequacy of security services. The second section was the Maslach Burnout Inventory (MBI) to assess the three burnout dimensions. The English version of the Maslach Burnout Inventory Human Services Survey"MBI-HSS"questionnaire [21] was translated into Arabic and back-translated into English. A comparison of the two versions was made to ensure the accuracy of the translation of questions. The content validity of the translated version was assessed by five public health and nursing professors, resulting in a content validity index (CVI) of 9.3. Cronbach's Alpha was 0.753 for the EE subscale, 0.750 for the DP subscale, and 0.793 for the PA subscale indicating high reliability. A pilot study on 20 nurses was conducted over a week to ensure clarity of the items and to identify any potential issues or challenges. No major changes were made. The pilot sample was not included in the study analysis. Ten minutes were needed to complete the questionnaire.

The MBI-HSS comprises 22 self-completed items that evaluate the presence of burnout over three dimensions: emotional exhaustion (nine items), depersonalization (five items), and sense of personal accomplishment (eight items). A 7-level Likert-type scaling method is applied to score the frequency of each of the 22 items, from score 0 (never) to score 6 (daily). The EE and DP subscales are graded similarly, while the PA subscale is an inverse one, as it is negatively phrased. The scores for each subscale are added. As a general principle, a high EE score (maximum score of 54), high DP score (maximum score of 30), and a low PA score (maximum score of 48) indicate burnout. The cut-offs being used to define the degree (high, average, or low corresponding to each dimension of burnout are as follows [21]:

- EE: high ≥ 27, average: 26-17, and low ≤ 16.
- DP: high ≥ 13, average: 12-7, and low ≤ 6; and
- PA: high ≤ 31, average: 32–38, and low ≥ 39.

### Data analysis

Data was analyzed using the IBM SPSS software package version 20.0. (Armonk, NY: IBM Corp). Qualitative data were described using numbers and percentages. The Kolmogorov–Smirnov test was used to verify the normality of distribution. Quantitative data was described using mean and standard deviation. The chi-square test was used to compare categorical variables between different groups. Student t-test and Analysis of Variance (ANOVA) were used for normally distributed quantitative variables. In the multiple linear regression models, the dependent variables (continuous variables) were EE, DP, and PA. The independent variables were socio-demographic and work-related characteristics. Only the variables that were significant concerning the three outcome variables separately in the bivariate analysis were included in the multivariate model. The independent variables included: gender, marital status, children, education level, income adequacy, job title, service area, type of shift, adequacy of physicians’ number, and providing care to COVID-19 patients. The reference categories were female, being married, having a child or more, diploma and technical nurse education level, earning an adequate income, working as a patient care nurse, working in a critical care unit, reporting adequate physicians’ number, and providing care to COVID-19 patients. Because the independent variables had low prediction power for the personal accomplishment subscale (< 10%), that analysis was not included in the results section. A *p* value < 0.05 was used to indicate statistical significance.

## Results

The mean age of the study sample was 35.31 ± 9.87 years, ranging from 22.0 to 59.0 years. The majority were females (83.4%), married (64.4%) and had children (66.8%). The highest percentage of education degrees was Bachelor's (42.3%) followed by technical degrees (28.6%), diplomas (21.6%), and postgraduate degrees (7.5%). The income was considered inadequate by 70.4% of the sample, and the mean and SD of the years of experience was 14.10 ± 10.16, ranging from 0.50–39.0 years (Table 1).

**Table 1:** Sociodemographic characteristics of nurses working in COVID-19 isolation hospitals in Alexandria, Egypt, 2022

Socio demographic characteristics	Total (*n* = 385)	
	**No.**	**%**
Age:		
20–30	163	42.3
> 30–40	108	28.1
> 40–50	73	19.0
> 50	41	10.6
Mean ± SD	35.31 ± 9.87	
Range	22.0–59.0	
Sex		
Male	64	16.6
Female	321	83.4
Marital status		
Married	248	64.4
Single/Divorced/Widowed	137	35.6
Children		
No children	128	33.2
≥ 1 children	257	66.8
Level of education		
Diploma	83	21.6
Technical	110	28.6
Bachelor’s degree	163	42.3
Postgraduate degree	29	7.5
Income		
Adequate	114	29.6
Less than adequate	271	70.4
Years of experience		
≤ 10	180	46.8
> 10–20	95	24.7
> 20–30	80	20.8
> 30	30	7.8
Mean + SD	14.10 ± 10.16	
Range	0.50–39.0	

Regarding the work-related characteristics, 80% were patient care nurses, and 17.9% had an administrative position. Most nurses worked in the critical care units (43.6%), and the inpatient department (35.6%), while only 11.4% worked in the emergency department and 3.6% in the outpatient department. Regarding the work time shift, 45.7% had rotating shifts, 21% had long day shifts, 17.7% worked morning shifts, and 14.5% worked night shifts. More than half of the nurses (52.2%) worked with a nurse-to-patient ratio of > 1:3, followed by 23.1% who worked with a ratio of 1:3 and 19.5% with a ratio of 1:2. The majority of the nurses (66.8%) provided direct care to COVID-19 patients. Only 44.7% reported that the number of physicians was adequate, 72.8% reported adequacy of the protective equipment, and 65.7% reported inadequate security services (Table 2).

**Table 2:** Work-related variables of nurses working in COVID-19 isolation hospitals in Alexandria, Egypt, 2022

Work-related variables	Total (*n* = 385)	
	**No.**	**%**
Job title		
Patient care nurse	308	80.0
Nursing Supervisor/manager	69	17.9
Quality/infection control/occupational	8	2.1
Service area		
Critical care unit	168	43.6
Emergency department	44	11.4
Inpatient department (surgical/medical/etc.)	137	35.6
Outpatient	14	3.6
Hospital management	22	5.7
Time of Shift		
Morning shift	68	17.7
Afternoon shift	4	1.0
Night shift	56	14.5
Rotating	176	45.7
Long day shift	81	21.0
Adequacy of physicians’ number		
Yes	172	44.7
No	213	55.3
Nurse to patient Ratio		
NA	7	1.8
1:1	13	3.4
1:2	75	19.5
1:3	89	23.1
> 1:3	201	52.2
Care provided to COVID patients		
Yes	257	66.8
No	128	33.2
Adequate protective equipment	(*n* = 257)	
Yes	187	72.8
No	70	27.2
Adequacy of security service		
Yes	132	34.3
No	253	65.7

Most of the nurses (82.3%) had high levels of EE with a mean score of 35.43 ± 9.52. DP was high among 60.8% with a mean score of 13.63 ± 5.81. The mean score of PA was 33.70 ± 7.43, and 34.5% of nurses had a low level of PA **(**Table 3**)**.

**Table 3:** Maslach burnout subscales among nurses working in COVID-19 isolation hospitals in Alexandria, Egypt, 2022

Burnout subscale	Total (385)	
	**No.**	**%**
Emotional exhaustion (EE)		
High ≥ 27	317	82.3
Average 26–17	54	14.0
Low ≤ 16	14	3.6
Total score (mean ± SD)	35.43 ± 9.52	
Depersonalization (DP)		
High ≥ 13	234	60.8
Average 12–7	99	25.7
Low ≤ 6	52	13.5
Total score (mean ± SD)	13.63 ± 5.81	
Personal accomplishment (PA)		
Low ≤ 31	133	34.5
Average 32–38	156	40.5
High ≥ 39	96	24.9
Total score (mean ± SD)	33.70 ± 7.43	

*SD* Standard deviation

Younger age was significantly associated with higher EE and DP and lower PA (F = 28.468, *p* < 0.001), F = 10.519, *p* < 0.00, F = 3.607, *p* = 0.014, respectively). Males had a statistically significant higher EE mean (38.13 ± 7.77) compared to females (34.90 ± 9.75) (*p* = 0.005). Being married was significantly associated with a lower level of EE (t = 3.454, *p* = 0.001) and DP (t = 4.212, *p* < 0.001). Having children was also significantly associated with lower values for all burnout dimensions (t = 6.222, *p* < 0.001, t = 5.201, *p* < 0.001, t = 3.403, *p* = 0.001, respectively). A higher education level had a significant association with a higher level of all burnout dimensions. Having adequate income was significantly associated with lower EE and DP (t = 2.482, *p* = 0.014 and t = 2.137, *p* = 0.033, respectively). A higher number of years of work was significantly associated with a lower level of all burnout dimensions (EE t = 41.106, *p* < 0.001, DP t = 15.781, *p* < 0.001, PA t = 3.995, *p* = 0.008, respectively) (Table 4)**.**

**Table 4:** Distribution of burnout dimensions according to sociodemographic characteristics among nurses working in COVID-19 isolation hospitals in Alexandria, Egypt, 2022

**Socio demographics**	Total (*n* = 385)		
	**EE**	**DP**	**PA**
	**Mean ± SD**	**Mean ± SD**	**Mean ± SD**
Age			
20–30	39.69 ± 7.47	14.99 ± 5.57	32.36 ± 7.51
>30–40	34.44 ± 8.64	13.69 ± 5.76	34.10 ± 7.19
>40–50	31.53 ± 9.29	12.71 ± 5.60	35.01 ± 7.25
>50	28.10 ±11.38	9.71 ± 5.37	35.61 ± 7.30
F (*p*)	(<0.001^*^)	(<0.001^*^)	(0.014^*^)
Gender			
Male	38.13 ± 7.77	14.52 ± 5.63	32.81 ± 8.06
Female	34.90 ± 9.75	13.45 ± 5.84	33.88 ± 7.29
t (*p*)	(0.005^*^)	(0.182)	(0.296)
Marital status			
Married	34.21 ± 9.78	12.76 ± 5.99	34.12 ± 7.71
Not married	37.66 ± 8.64	15.20 ± 5.14	32.93 ± 6.84
t (*p*)	0.001^*^	<0.001^*^	0.133
Children			
No children	39.23 ± 7.68	15.74 ± 5.10	31.90 ± 7.12
≥1 children	33.54 ± 9.79	12.58 ± 5.87	34.60 ± 7.42
t (*p*)	<0.001^*^	<0.001^*^	0.001^*^
Level of education			
Diploma	30.01 ±10.05	12.71 ± 5.55	34.23 ± 7.66
Technical	37.87 ± 6.99	15.17 ± 5.42	32.63 ± 6.19
Bachelor	36.85 ± 9.49	13.35 ± 6.00	33.59 ± 8.01
Postgraduate	33.72 ±10.50	11.97 ± 5.93	36.86 ± 6.94
F (*p*)	<0.001^*^	0.005^*^	0.045^*^
Income			
Adequate	33.84 ± 9.83	12.41 ± 6.56	34.03 ± 8.28
Less than adequate	36.10 ± 9.32	14.14 ± 5.40	33.56 ± 7.05
t (*p*)	0.033^*^	0.014^*^	0.575
Years of experience			
≤10	39.63 ± 7.51	15.34 ± 5.54	32.42 ± 7.42
>10–20	34.45 ± 8.09	13.12 ± 5.21	34.32 ± 7.24
>20–30	31.48 ± 9.35	12.24 ± 5.87	34.88 ± 6.68
>30	23.90 ±10.41	8.70 ± 5.20	36.30 ± 8.79
F (*p*)	<0.001^*^	<0.001^*^	0.008^*^

*SD* Standard deviation, t: Student t-test F: F for One-way ANOVA test

*p: p*-value for comparison between the studied categories

*Statistically significant at *p* ≤ 0.05

Studying the association between work-related factors and burnout dimensions showed that nurses having managerial or supervision roles scored significantly lower on the EE (F = 5.951, *p* = 0.003), DP (F = 7.323, *p* = 0.001), and significantly higher on the PA dimension (F = 5.766, *p* = 0.003). Regarding the service area, the highest EE was among the critical care unit (mean ± SD = 37.70 ± 9.50), while the highest DP was among inpatient services nurses (mean ± SD = 14.66 ± 5.42) and the differences were significant. The morning time of the shift was significantly associated with the lowest EE (mean ± SD = 29.24 ± 10.30). The highest DP mean was among the afternoon shift nurses (16.00 ± 6.22). For personal accomplishment, morning shift nurses had the highest mean (35.96 ± 6.29), and the differences were significant. The adequacy of the number of physicians was significantly associated with lower EE and DP and higher PA (33.07 ± 9.84, 12.12 ± 5.92, 34.92 ± 6.90, respectively). Nurses providing direct care to COVID-19 patients had significantly higher EE (36.41 ± 9.34). The adequacy of security services was significantly associated with lower EE and DP (33.73 ± 10.26 and 11.55 ± 6.25) and the difference was significant (Table 5).

**Table 5:** Distribution of burnout dimensions according to work-related characteristics among nurses working in COVID-19 isolation hospitals in Alexandria, Egypt, 2022

Organizational variables	Total (*n* = 385)		
	**EE**	**DP**	**PA**
	**Mean ± SD**	**Mean ± SD**	**Mean ± SD**
Job title			
Patient care nurse	36.25 ± 9.21	14.19 ± 5.72	33.13 ± 7.46
Supervisor/manager	31.96 ± 10.23	11.46 ± 5.71	36.41 ± 6.63
Quality/infection control/occupational unit	34.13 ± 9.34	10.88 ± 5.96	32.38 ± 8.37
F (*p*)	0.003^*^	0.001^*^	0.003^*^
Service area			
CCU	37.70 ± 9.50	13.49 ± 5.87	33.46 ± 8.22
ED	33.00 ± 9.36	12.20 ± 6.59	32.48 ± 8.32
Inpatient unit	34.72 ± 8.89	14.66 ± 5.42	34.05 ± 6.24
Outpatient department	29.21 ± 8.96	13.57 ± 4.59	32.93 ± 3.65
Hospital management	31.45 ± 9.92	11.18 ± 5.84	36.23 ± 7.58
F (*p*)	< 0.001^*^	0.027^*^	0.360
Time of Shift			
Morning	29.24 ± 10.30	11.32 ± 5.04	35.96 ± 6.29
Afternoon	34.75 ± 12.04	16.00 ± 6.22	29.75 ± 6.13
Night	36.71 ± 9.09	15.88 ± 5.99	32.48 ± 7.64
Rotating	37.70 ± 8.66	14.36 ± 5.63	33.04 ± 7.38
Long day	34.85 ± 8.62	12.31 ± 5.85	34.27 ± 7.97
F (*p*)	< 0.001^*^	< 0.001^*^	0.028^*^
Adequate number of physicians			
Yes	33.07 ± 9.84	12.12 ± 5.92	34.92 ± 6.90
No	37.34 ± 8.82	14.85 ± 5.44	32.71 ± 7.70
t (*p*)	< 0.001^*^	< 0.001^*^	0.004^*^
Nurse: patient Ratio			
NA	27.29 ± 12.91	11.00 ± 4.62	35.71 ± 7.80
1:1	31.54 ± 9.39	11.85 ± 6.68	33.00 ± 6.06
1:2	34.99 ± 8.99	13.59 ± 5.35	33.28 ± 8.63
1:3	36.10 ± 9.86	12.96 ± 5.98	33.39 ± 8.65
> 1:3	35.84 ± 9.35	14.15 ± 5.86	33.97 ± 6.40
F (*p*)	0.081	1.371(0.243)	0.317(0.866)
Provides care to COVID patients			
Yes	36.41 ± 9.34	13.30 ± 5.95	33.91 ± 7.57
No	33.47 ± 9.61	14.29 ± 5.50	33.27 ± 7.13
t (*p*)	0.004^*^	0.116	0.428
Adequate protective equipment			
Yes	33.73 ± 10.26	11.55 ± 6.25	34.17 ± 7.60
No	36.32 ± 9.01	14.71 ± 5.27	33.45 ± 7.33
t (*p*)	0.015^*^	< 0.001^*^	0.365
Adequacy of security service			
Yes	33.73 ± 10.26	11.55 ± 6.25	34.17 ± 7.60
No	36.32 ± 9.01	14.71 ± 5.27	33.45 ± 7.33
t (*p*)	0.015^*^	< 0.001^*^	0.365

*SD* Standard deviation, t Student t-test, *F* F for One-way ANOVA test

CCU Critical Care Unit*, ED* Emergency Department

*p*: *p*-value for comparison between the studied categories

^*^Statistically significant at p ≤ 0.05

The multiple regression analysis of demographic and work characteristics showed that nurses having no children, nurses with Bachelor degrees, those with postgraduate studies, and those who reported inadequacy of the number of physicians in their units had significantly higher odds (3.98, 2.37, and 3.25, respectively) of higher emotional exhaustion (EE) scores compared to those who have children, nurses with technical or diploma nursing degree and those who perceived physician number as adequate (Confidence interval (CI): 1.122—6.853, 1.203 −3.551, 1.425 −5.087, respectively). On the other hand, nurses working morning shifts had significantly lower odds of reporting higher EE scores (0.904) compared to those working rotating shifts (CI:0.390–0.947). The predictive model explained 24.0% of the variance in emotional exhaustion, and the model is statistically significant (F = 6.8, *p* < 0.001) (Table 6)**.**

**Table 6:** Multivariate Linear regression of sociodemographic and work characteristics affecting emotional exhaustion among nurses

Independent variable	B	Beta	t	Sig	95% Confidence Interval for B	
					**Lower**	**Upper**
Gender [Female ®]	0.37	0.15	0.29	0.77	0.166	2.924
Marital status [Married ®]	0.50	0.02	0.37	0.71	0.160	3.167
Children [≥ 1 children ®]	3.98	0.19	2.73	0.00*	1.122	6.853
Education [Technical/Diploma ®]	2.37	0.22	3.98	0.00*	1.203	3.551
Income [Adequate ®]	2.04	0.09	1.95	0.05	0.015	4.098
Job title						
Patient care nurse ®						
Nursing Supervisor/manager	2.26	1.55	−0.09	0.14	0.801	5.326
Quality/infection control/occupational	0.13	0.00*	0.03	0.97	0.076	8.344
Service area						
Critical Care Unit ®						
Emergency department	2.69	0.09	1.81	0.07	0.225	5.623
Inpatient department	0.24	0.01	0.22	0.82	0.948	2.438
Outpatient department	1.72	0.03	0.58	0.55	0.050	7.507
Hospital management	2.40	0.05	0.91	0.35	1.561	2.748
Type of Shift						
Rotating®						
Morning shift	0.904	0.17	3.11	0.04*	0.390	0.947
Afternoon shift	3.71	0.04*	0.8	0.40	0.969	1.239
Night shift	0.70	0.02	0.52	0.60	0.461	3.378
Long day shift	1.36	0.05	1.10	0.27	0.073	3.802
Adequacy of physician number®	3.25	0.17	3.49	0.00*	1.425	5.087
Care provided to COVID patients®	1.77	0.08	1.76	0.07	0.200	3.753

® Reference category

*R*^2^ = 24.0%, F = 6.802, *p* = 0.00

For depersonalization, the multiple regression analysis of demographic and work characteristics showed that nurses not having children and those who reported inadequacy of the number of physicians in their units had significantly higher odds (2.4 and 2.2) of having high depersonalization score compared to those who have children or perceive the number of physicians to be adequate (CI: 1.676—4.273, 1.083—3.374, respectively). Similarly, nurses working in the inpatient and outpatient departments have higher odds (2.1 and 3.6) of reporting higher depersonalization scores than nurses in the critical care units (CI: 1.790—3.504, 2.053—7.211, respectively). The predictive model explained 15.8% of the variance in depersonalization and the model is statistically significant (F = 5.8, *p* < 0.00)(Table 7).

**Table 7:** Multivariate Linear regression of sociodemographic and work characteristics affecting depersonalization among nurses

Independent variables	B	Beta	t	Sig	95% Confidence Interval for B	
					**Lower**	**Upper**
Marital status [Married ®]	0.697	0.02	3.49	0.727	0.374	1.968
Children [≥ 1 children ®]	2.475	0.20	2.70	0.007^*^	1.676	4.273
Education [Technical/Diploma ®]]	0.509	0.07	1.37	0.171	0.220	1.237
Income [Adequate ®]	1.053	0.08	1.61	0.107	0.227	2.333
Job title						
Patient care nurse ®						
Nursing Supervisor/manager	0.769	0.04	0.69	0.349	0.371	2.575
Quality/infection control/occupational	0.231	0.02	0.43	0.679	0.015	1.261
Service area						
Critical Care Unit ®						
Emergency department	0.381	0.02	0.41	0.276	0.148	2.211
Inpatient department	2.147	0.17	3.11	0.002^*^	1.790	3.504
Outpatient department	3.632	0.11	1.99	0.047^*^	2.053	7.211
Hospital management	0.721	0.00	0.07	0.941	0.308	3.327
Type of Shift						
Rotating®						
Morning shift	0.859	0.12	1.77	0.076	0.198	3.916
Afternoon shift	1.422	0.02	0.51	0.605	0.816	3.973
Night shift	1.418	0.08	1.69	0.090	0.223	3.059
Long day shift	1.102	0.07	1.41	0.157	0.425	2.630
Adequacy of physicians®	2.228	0.19	3.82	< 0.001*	1.083	3.374

® Reference category, *R2* 15.8%, *F *5.81, *P* 0.00

## Discussion

The COVID-19 pandemic has had a great impact on the healthcare system around the world. Nurses, who are at the frontlines of healthcare responses, have been particularly affected by the pandemic. In this study, nurses reported suffering from high levels of burnout; with a high level of EE among the majority (82.3%), a high level of DP among about two-thirds of nurses (60.8%) and a high level of PA among only 24.9% of the total sample.

Levels of burnout increased among nurses during the COVID-19 pandemic. The degree of burnout among nursing staff working in a university hospital in Egypt measured using the MBI before the pandemic showed that 43.2% were classified as having a moderate level of burnout and 32.6% as having a high level of burnout in 2016 [9] On the other hand, during the COVID-9 crisis it was shown that more nurses scored high in EE and DP subscales (86% and 52%) in 2020 [19]. 

Nurses who work in COVID-19-designated hospitals or units were found to have high levels of burnout in Turkey [22] and China [23]. In Egypt, a study investigating stress among nurses in isolation hospitals during COVID-19 found that 52.1% of the studied nurses had a moderate level and 26.2% had a severe level of stress [24]. This is comparable to the current study’s results. This higher level of burnout can be explained by the high stress of working conditions in the isolation hospitals during COVID-19. Both hospitals were assigned to be isolation hospitals for COVID-19 cases during the time of the study, which may have played a role in this high level of burnout. The pandemic inflicted pressures of different kinds on all components of the healthcare system including the healthcare providers of which the nurses were mostly affected. 

Other research showed high levels of burnout among nurses during COVID-19. In a systematic review of 16 studies comprising 18,935 nurses, significant levels of burnout were reported (34.1%, 15.2%, and 12.6% of nurses experienced high levels of emotional exhaustion, low personal accomplishment and depersonalization respectively) [25]. Another systematic review of 113 studies and meta-analysis of 61 studies comprising 45,539 nurses worldwide in 49 countries across multiple specialties showed high levels of burnout during the COVID-19 pandemic (an overall pooled-prevalence of burnout symptoms among nurses was 11.23%) [26]. Higher workload during COVID-19, inadequate training, the stress imposed by the long hours of work and the great number of cases at that time were all significant contributing factors that profoundly impacted nurses [25].

Among the sociodemographic and personal factors, younger age, being unmarried, not having offspring, a technical and bachelor level of education, insufficient income, and a lower number of work years were all significantly associated with higher levels of the burnout dimensions. The regression model revealed that not having children predicted higher levels of both EE and DP, and higher level of education degree predicted higher EE. Although having children may be seen as a significant load on nurses, especially during the pandemic with the ensuing fear of transmitting the infection to them as well as the inadequate time to take care of their offspring due to the increasing work hours, it was found to have a relieving effect. This may be explained in our case by the possible association of having children with older age, which was found to have a negative association with burnout. Additionally, having children often coincides with a higher likelihood of being married, which was also significantly associated with lower EE and DP scores. Moreover, Nurses with children may develop better coping strategies due to their familial responsibilities, while work-life balance may be more strained for single nurses, leading to higher emotional distress. Higher education levels of nurses may be accompanied by higher responsibilities and higher ambition to excel in the job. Besides, highly educated persons may have more worries about their future.

Literature showed inconsistent findings regarding the possible predictors or associated factors of burnout syndrome dimensions. For example, age was shown to have a variable relation to burnout. It has been reported in some studies that younger age was associated with higher burnout [22, 25, 27] while other studies found a significant relationship between older age and higher burnout score [13, 28]. Older age accompanied by a longer duration of work years was found to have a significant relationship with burnout [20]. Sociodemographic characteristics such as income [19, 28], higher level of education [22, 29, 30], marital status [20, 28], and having children [29, 28] were found to be predictors of at least one dimension of burnout. In the current study, gender was associated with emotional exhaustion where males had higher mean scores than females (38.13 ± 7.77, 34.90 ± 9.75, t = 2.900, *p* <0.05). Similarly, a meta-analysis of 57 studies in 2018 found the male gender to be associated with higher burnout [31]. However, another systemic review found no relation to gender [25]. 

A significant association of the work-related variables with the burnout dimensions levels among nurses was found. Being a patient care nurse, shift times other than morning (especially night) and reporting an inadequate number of physicians were significantly associated with higher burnout subscales scores, while providing direct care to COVID-19 patients, working in the inpatient department, and inadequacy of personal protective equipment and security services were more significantly associated with EE for the first variable and both EE and DP for the latter two variables. Nurses who had managerial or supervisory duties in this study were found to have significantly less EE and DP and higher PA. The regression model showed that those working in the inpatient and outpatient departments are predicted to have higher DP scores. Working morning shifts predicted lower EE, while perceiving inadequacy of physicians’ numbers predicted both burnout dimensions.

Fear of infection and inadequate personal protective equipment were positive predictors of stress in COVID-19 isolation hospitals in China [29]. The perceived inadequate human and material resources were also significantly associated with burnout among nurses during COVID-19 in Spain [32]. These results are similar to the current study. Studying burnout during COVID-19 in Saudi Arabia showed that inadequate security and lack of managerial position were predictive factors for at least one dimension of burnout [30]. Performing administrative tasks was associated with less DP and higher PA among nurses in public health centers in Spain [28].

Inpatient and outpatient nurses may face higher patient loads and more administrative burdens than critical care nurses, who may have better staffing ratios and team support. Physician shortages may increase nurses'responsibilities, leading to greater physical and emotional exhaustion. Also, they may feel unsupported in professional decision-making during work. Night shifts may disrupt circadian rhythms, impair sleep quality, and lead to chronic fatigue and burnout. Moreover, the family responsibilities of nurses may be disturbed due to their absence from homes at a time when no assistance can be obtained. During the pandemic, social distancing was an obstacle against using house helpers to control the spread of the virus. One of the major factors that enhances safety among healthcare workers is the use of adequate amounts of personal protective equipment as well as their quality. The deficiency of PPE may leave them insecure and worried about exposure to infection. Being involved in decision making and having control over one’s job seems to offer some sort of protection against burnout.

### Limitations of the study

The study setting was limited to two isolation hospitals only. Although large sized, it is not enough to generalize the results to all other hospitals’ nurses inside and outside Alexandria. In addition, a cross-sectional design was used, which does not allow inference of causality. Using a self-reported tool may predispose the results to recall and social desirability bias.

## Conclusion

There is a high burnout level among nurses working in COVID-19 isolation hospitals. Predictors of EE and DP include having no children, and the nurse’s perception of an inadequate number of physicians. As nurses providing direct patient care to COVID-19 patients are vulnerable to high levels of burnout, it is recommended to support them with planned training on coping with the relevant stresses, and psychological support services whenever needed. Social services that support the nurses may also help to overcome the stress imposed by the long work hours. The administrative authorities should review the work-related factors such as the inadequacy of the physicians’ number and insufficient security services. Work procedures should be developed for late shifts to ensure adequate staffing levels and implementation of regular shift rotations to alleviate the negative effects of prolonged late shifts. Adequate rest following such shifts can also help.

## Data Availability

The data set for the current study is available from the corresponding author upon a reasonable request.

## Abbreviations

COVID-19: Coronavirus disease of 2019
MBI: Maslach Burnout Inventory
EE: emotional exhaustion
DP: depersonalization
PA: personal accomplishment
(ANOVA): Analysis of Variance
SD: Standard deviation
CCU: Critical Care Unit
ED: Emergency Department
PPE: Personal Protective Equipment
